# Anxiety and depression in children and adults: influence of serotonergic and neurotrophic genes?

**DOI:** 10.1111/j.1601-183X.2010.00619.x

**Published:** 2010-10

**Authors:** C M Middeldorp, M C T Slof-Op 't Landt, S E Medland, C E M van Beijsterveldt, M Bartels, G Willemsen, J-J Hottenga, E J C de Geus, H E D Suchiman, C V Dolan, M C Neale, P E Slagboom, D I Boomsma

**Affiliations:** † Department of Biological Psychology, VU University AmsterdamAmsterdam; ‡ De Bascule, Academic Center for Child and Adolescent PsychiatryAmsterdam; § Molecular Epidemiology, Leiden University Medical CenterLeiden, The Netherlands; ¶ Genetic Epidemiology Unit, Queensland Institute of Medical ResearchBrisbane, Australia; ** Virginia Institute of Psychiatric and Behavioral Genetics, Virginia Commonwealth UniversityRichmond, VA, USA; †† Department of Psychology, University of AmsterdamAmsterdam, The Netherlands

**Keywords:** Anxiety, cell signaling, depression, genetics, neurogenesis, serotonin

## Abstract

There are two major hypotheses regarding the etiology of anxiety and depression: the mono-amine hypothesis and the hypothesis of an abnormal stress response acting partly via reduced neurogenesis. Association studies have focused on genes involved in these processes, but with inconclusive results. This study investigated the effect of 45 single nucleotide polymorphisms (SNPs) in genes encoding for serotonin receptors 1A, 1D, 2A, catechol-O-methyltransferase (COMT), tryptophane hydroxylase type 2 (TPH2), brain derived neurotrophic factor (BDNF), PlexinA2 and regulators of G-protein-coupled signaling (RGS) 2, 4, 16. Anxious depression (A/D) symptoms were assessed five times in 11 years in over 11 000 adults with 1504 subjects genotyped and at age 7, 10, 12 and during adolescence in over 20 000 twins with 1078 subjects genotyped. In both cohorts, a longitudinal model with one latent factor loading on all A/D measures over time was analysed. The genetic association effect modeled at the level of this latent factor was 60% and 70% heritable in the children and adults, respectively, and explained around 50% of the total phenotypic variance. Power analyses showed that the samples contained 80% power to detect an effect explaining between 1.4% and 3.6% of the variance. However, no SNP showed a consistent effect on A/D. To conclude, this longitudinal study in children and adults found no association of SNPs in the serotonergic system or core regulators of neurogenesis with A/D. Overall, there has been no convincing evidence, so far, for a role of genetic variation in these pathways in the development of anxiety and depression.

The genetic etiology of anxiety disorders and depression has been investigated extensively. Most studies have used a candidate gene approach to identify relevant genetic variants. In these studies, the investigated genes were usually chosen because of their involvement in neurotransmission circuits, or in the stress response and related processes such as neurogenesis, because malfunctions in these biological pathways are most commonly hypothesized to play a role in the development of anxiety and depression (Belmaker & Agam 2008). Despite the wealth of studies, results to date have been inconclusive (Levinson 2006; Stoppel *et al.* 2006). There may be several reasons for this lack of definite result. Most studies lacked power to detect the small effects of these polymorphisms (Lohmueller *et al.* 2003). In addition, the effect of the implicated genes may depend on the age, sex or ethnicity (Verhagen *et al.* 2010) and on exposure to adverse (life) events (Moffitt *et al.* 2005).

Statistical power can be increased by pooling single studies in meta-analyses (Levinson 2005). However, as Munafo *et al.* (2005) pointed out, ‘A meta-analysis can only be as good as the individual studies that contribute to it’ (p. 896), and therefore, ‘Very large, well-designed studies remain the most reliable way of obtaining reproducible results’ (p. 897). Power can also be increased by using a longitudinal design (Hottenga & Boomsma 2007). This can be a valuable strategy in genetic association studies on anxiety and depression, as genetic factors influencing these symptoms appear to be moderately stable from childhood to at least 20 years of age (Boomsma *et al.* 2007; Kendler *et al.* 2008). Longitudinal studies in adults also show that stability in anxiety and depression is largely attributable to genetic factors (Gillespie *et al.* 2004; Nes *et al.* 2007; Rijsdijk *et al.* 2003).

We present a genetic association study of longitudinal data on anxiety and depression (A/D) symptoms collected in two large samples of children and adults. In over 11 000 adult twins, siblings and parents, data were collected at five time points in approximately 11 years. In over 20 000 twins, data were collected atages 7, 10, 12 and during adolescence. In both cohorts, we first modeled the longitudinal data with a one-factor model to explain the covariance between measures. In subsamples of over 1500 adults and over 1000 children, genotype data consisting of single nucleotide polymorphisms (SNPs) were assessed. The effect of the SNPs was modeled on the common factor, thereby making optimal use of the available data.

Forty-five SNPs were assessed in candidate genes that were involved in (1) the mono-aminergic system: serotonin receptors (HTR) 1A, 1D, 2A, catechol-O-methyltransferase (COMT), tryptophane hydroxylase type 2 (TPH2), (2) neurogenesis: brain derived neurotrophic factor (BDNF) and PlexinA2 and (3) cell signaling: regulators of G-protein signaling (RGS) 2, 4, 16.

The effect of the SNPs was allowed to differ between men and women as previous meta-analyses have suggested sex-specific effects for several genes (e.g. COMT Val158Met and BDNF Val66Met polymorphisms) (Domschke *et al.* 2007; Verhagen *et al.* 2010).

## Materials and methods

### Subjects

#### Adult sample

The data come from the longitudinal survey studies of the Netherlands Twin Register (NTR) that has assessed families with adolescent and adult twins roughly every 2 years since 1991. Sample selection and response rates are described in detail in Boomsma *et al.* (2002, 2006). Data from twins, parents and siblings collected during the surveys in 1991, 1995, 1997, 2000 and 2002 were analysed. Twins were approached to participate at all occasions. Parental data were collected in 2002 and sibling data in 1997, 2000 and 2002.

Subjects who were aged between 18 and 65 at the time of the assessment and twins with known zygosity were included. Data from half siblings were excluded, because these are scarce. For same-sex twin pairs, zygosity was determined from survey items or DNA polymorphisms (726 twin pairs). The agreement between zygosity diagnoses from questionnaire and DNA data was 97% (Willemsen *et al.* 2005).

Subsamples of the twin-families were invited to participate in experimental and laboratory studies and provide a DNA sample (Boomsma *et al.* 2006). Genotyping data were obtained for 1943 subjects with at least one A/D score available for 562 men and 942 women. As there were 84 monozygotic male (MZM) and 178 monozygotic female (MZF) twins, 562 − 84/2 = 520 unique male and 942 − 178/2 = 853 unique female genotypes were included in the analysis. The SNPs in RGS2, 4 and 16 were genotyped in a smaller sample. The final sample, in which the effect of the RGS SNPs was analysed, consisted of 321 men and 489 women, including 20 MZM and 42 MZF twins.

Longitudinal modeling of data was first carried out using data from all 11 516 twins, parents and siblings (with a maximum of one brother and one sister per family) from 4427 families. This sample consisted of 1145 MZM twins, 797 dizygotic male (DZM) twins, 2323 MZF twins, 1323 dizygotic female (DZF) twins and 829 male and 987 female dizygotic twins of opposite sex (DOS) and 1116 fathers, 1376 mothers, 715 brothers and 905 sisters.

#### Child and adolescent sample

Young twins are registered with the NTR at birth by their parents. During the first 12 years of their lives the parents are the primary sources of information on the twins' development. After age 14, twins and their siblings receive a self-report survey by mail (Bartels *et al.* 2007). Parental data collected at ages 7, 10 and 12 were included in this study plus one self-report measure collected between age 14 and 18.

Subsamples of the young twins were also invited to participate in experimental and laboratory studies and provide a DNA sample (Boomsma *et al.* 2006). Genotyping data were obtained for 1240 subjects which included 526 boys and 552 girls who also had at least one A/D score. As this sample included 288 MZM and 320 MZF twins, there were 382 unique male and 392 unique female genotypes.

Similar to the procedure in the adult sample, longitudinal modeling was carried out first in the total sample consisting of 20 414 twins from 10 227 families, who participated at least once, including 3318 MZM, 3443 DZM, 3796 MZF, 3249 DZF, 3300 male and 3308 female DOS twins.

### Measures

In the adult sample, A/D was measured with the anxious depression scale of the Young Adult Self-Report (YASR) (Achenbach 1990). For the young twins, paternal and maternal ratings of the A/D scale of the Child Behavior Check List (CBCL) (Achenbach 1991; Verhulst *et al.* 1996) were available at ages 7, 10 and 12 and a self-report measure of the A/D scale of the Youth Self Report (YSR) (Verhulst *et al.* 1997) during adolescence. The YASR, CBCL and YSR belong to the Achenbach System of Empirically Based Assessment (ASEBA, http://www.aseba.org), which provides age-adjusted instruments to assess similar facets of maladaptive functioning from 1.5 to 90 years. Thus, the A/D scales used in the children, adolescents and adults are very comparable.

To minimize skewness, A/D data of children and adults were normalized in the total samples with the option ‘NS’ in LISREL 7 (Jöreskog & Sörbom 1989).

### Statistical methods

**Figure 1:**
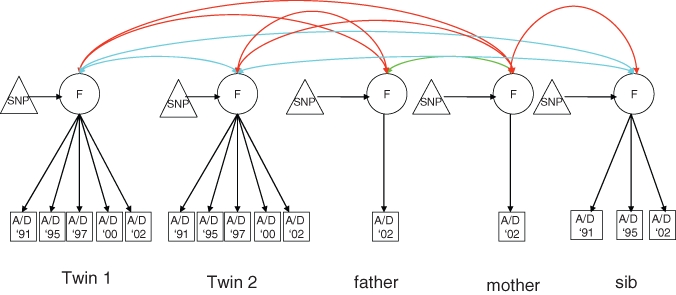
Factorial association model in the adults The latent A/D factor loads on the longitudinal A/D measures (5 in the twins, 1 in the parents and 3 in the siblings), reflecting the stability across time. The effect of the SNP is modeled on the A/D means through the A/D factor. The blue arrows reflect the correlation between twins and siblings. Twin correlations differ for MZ twin pairs and DZ twin pairs. The red arrows reflect the parent–offspring correlations. The green arrow reflects the parental correlations. All estimates are allowed to vary depending on sex. Not shown in the Figure (for the sake of clarity), but included in the model: the variance of the A/D factor, which is constrained to 1, the residual variances of the means and the effect of age on the mean.

**Figure 2:**
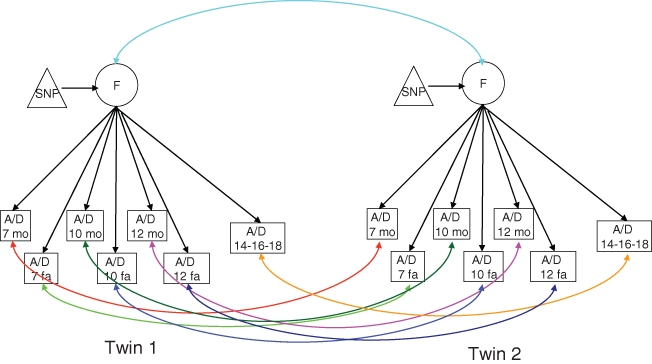
Factorial association model in the children The latent A/D factor loads on the longitudinal A/D measures (3 maternal (mo) and 3 paternal (fa) at age 7, 10 and 12 and one self-report at age 14, 16 or 18), reflecting the stability across time. The effect of the SNP is modeled on the A/D means through the A/D factor. The light blue arrows reflect the twin correlation. The other arrows reflect the twin correlations for the residuals to allow for age-dependent familial effects. The correlations differ for MZ and DZ twin pairs. All estimates are allowed to vary depending on sex. Not shown in the figure (for the sake of clarity), but included in the model: the variance of the A/D factor, which is constrained to 1, the latent residual factors and the effect of age on the mean of the adolescent measure.

Figures 1 and 2 show the models analysed to test the effect of the SNPs on A/D in adults and children, respectively, based on Medland and Neale (2010). One latent factor loads on all A/D measures and reflects the stability across time. From now on, we refer to this factor as the A/D factor. As A/D measures of members of the same family are not independent, the latent factors were correlated between relatives allowing different male and female monozygotic (MZ) twin, dizygotic (DZ) twin/sibling, father–son, father–daughter, mother–son, mother–daughter and parental correlations. Genetic epidemiological analyses in the children's sample have shown that genetic and shared environmental factors are not entirely the same over the ages (Boomsma *et al.* 2005). Therefore, in the model for the children's sample, the residual variances could also explain familial clustering expressed in a correlation between twins dependent on zygosity. As earlier twin studies have suggested sex differences in the variance–covariance structure, factor loadings and familial correlations were allowed to differ in men and women (Boomsma *et al.* 2000, 2005). Age and sex were included as covariates on the observed variables for the adults and the adolescents.

In the genetic association analyses, the additive effect of the SNPs was modeled through the A/D factor (Fig. 1). The effect of the SNP was also allowed to differ between men and women. For example, A/D measured in male adults in 1991 is:

A/D = *μ* + β_1_^*^age + β_2_^*^sex + β_3m_^*^f_m1991_^*^SNP, in which *f*_m1991_ is the factor loading from the A/D factor on A/D measured in 1991 in men, and the SNP genotype is coded as 0, 1 or 2 reflecting the number of minor alleles.

In the association analyses performed in the subsample with genotype data available, the factor loadings and residual variance terms in the models of Figs 1 and 2 were constrained at the estimates obtained in the total sample. The A/D factor correlations between relatives were freely estimated, because these vary as a function of the effect of the SNP on the latent factor.

Standard software for association analyses is not well equipped for such complex models, including both family and longitudinal data. Therefore, analyses were performed in Mx version 1.7, a software program designed for structural equation modeling (Neale *et al.* 2006). To use all data, analyses were performed on raw data using the full information maximum likelihood (FIML) method.

Analyses of the power to detect a genotypic effect in a multivariate model were also performed in Mx. *P*-values below 0.01 are reported. We decided not to use a more conservative correction of the *P*-value beforehand as the tests are not entirely independent because of linkage disequilibrium (LD) between SNPs in the same gene. Moreover, we did not want to overlook consistent patterns in results across samples because of a stringent correction for multiple testing as such patterns may point to genuine effects, e.g. several SNPs with a *P*-value below 0.01 in the same gene or in both the children's and adults' sample. The multiple testing problem will be considered in the interpretation of the results.

### Genotyping

Table 1 shows the genotyped SNPs. For most genes, the selection of SNPs was based on previous publications showing an association with depression and/or anxiety (Finn *et al.* 2003; Leygraf *et al.* 2006; Lopez-Leon *et al.* 2008; Wray *et al.* 2007). Additional tagging SNPs were selected for BDNF and TPH2 in order to capture most of the variation in these genes. SNPs in RGS4 and 16 were genotyped, because these genes lie in the same region as RGS2, which has been found to be associated with anxiety (Leygraf *et al.* 2006). This region has shown a highly significant linkage peak with emotionality in mice (Flint *et al.* 1995). Moreover, RGS4 has been implicated in schizophrenia (Chowdari *et al.* 2002), although results have not been conclusive (Li & He 2006). The expression of RGS16 is triggered by the same mitogen and cytokine receptors as RGS2 and 4. Tagging SNPs for BDNF and TPH2, were selected from HapMap Public Release #19, applying the efficient multimarker method with *r*^2^ > 0.8, and minor allele frequency (MAF) >0.05 as implemented in the HapMap web browsers (http://www.hapmap.org) (de Bakker *et al.* 2005). For the genomic region of the BDNF gene, we captured 38 of 53 (71%) alleles at *r*^2^ > 0.8. For the TPH2 gene we captured 108 of 148 (72%) alleles at *r*^2^ > 0.8. In addition, for the HTR1D gene we captured 8 of 10 (80%) alleles at *r*^2^ > 0.8. Figure S1 shows the LD plots for the TPH2, BDNF and PLXNA2 SNPs. The SNPs in the other genes were all in high LD (*D*’ is higher than 91 and in the majority of the SNPs higher than 95) with the exception of rs6311 and 6314 as well as rs 6314 and 6313 (*D*’ is 25 and 28, respectively).

**Table 1:** The genotyped SNPs per gene, MAF, the results of the HWE test in the total sample and the effect of the SNPs (β) in the male (m) and female (f) adult and children's sample. Printed in bold are the βs with a *P*-value <0.05

Genes	SNPs		MAF	*p*-Value HWE	βm adults	βf adults	βm children	βf children
HTR1A	rs6295	C<G	47.2	ns	0.11	−0.04	−0.08	0.07
HTR1D	rs605367	C<T	33.5	ns	0.15	−0.02	0.05	−0.02
	rs676643	A<G	15.6	ns	**0.29**	−0.06	−0.01	0.04
	rs2860192	G<A	8.8	ns	0.07	−0.01	0.08	0.09
	rs674386	A<G	30.2	ns	0.12	0.00	0.01	−0.03
HTR2A	rs6311	T<C	41.7	ns	−0.06	−0.12	0.02	−0.03
	rs6314	A<G	9.1	ns	0.10	−0.07	−0.03	−0.06
	rs6313	A<G	42.3	ns	0.05	−0.13	0.04	−0.08
COMT	rs4680	G<A	45.1	ns	−0.06	−0.03	−0.05	0.15
TPH2	rs1007023	G<T	14.1	0.0005	0.16	−0.00	−0.17	−0.02
	rs10748190	G<A	41.2	ns	−0.03	0.04	−0.10	−0.00
	rs12231356	T<C	6.7	ns	−0.29	−0.01	−0.02	−0.37
	rs1352251	C<T	40.8	ns	0.01	0.03	−0.11	−0.02
	rs1473473	C<T	15.8	0.00005	0.08	−0.06	−0.12	−0.00
	rs2129575	T<G	26.5	ns	−0.13	0.04	−0.03	0.12
	rs2171363	A<G	43.5	ns	0.03	0.06	−0.16	−0.02
	rs3903502	T<C	41.6	ns	−0.03	0.04	−0.10	0.00
	rs4474484	A<G	36.3	ns	−0.07	−0.01	−0.09	0.01
	rs4760820	G<C	40.5	ns	−0.05	−0.05	0.12	0.04
	rs7305115	A<G	43.4	ns	0.04	0.05	−0.16	−0.04
	rs10748185	A<G	47.8	ns	−0.03	0.05	−0.15	0.02
	rs17110489	C<T	27.4	ns	−0.00	0.00	−0.04	0.10
	rs7300641	T<G	16.7	0.0007	0.07	0.10	−0.21	−0.07
BDNF	rs2049048	A<G	15.5	ns	0.06	−0.12	−0.04	0.13
	rs7103873	C<G	45.5	ns	0.16	−0.01	−0.01	0.11
	rs6265	T<C	21.1	ns	−**0.26**	0.05	−0.05	−0.15
	rs11030107	G<A	25.2	ns	−0.06	0.07	0.06	−0.02
	rs11030123	A<G	10.2	ns	0.16	−0.10	−0.11	0.31
	rs1491851	T<C	44.5	ns	0.10	−0.09	0.06	0.04
	rs17309930	A<C	19.0	ns	0.06	0.06	0.06	−0.14
	rs7124442	C<T	30.6	ns	0.05	−0.01	0.08	−0.02
PLXNA2	rs2478813	A<G	12.4	ns	−0.02	−0.03	−0.08	−0.07
	rs3736963	G<A	41.5	ns	0.00	0.00	0.10	0.12
	rs752016	G<A	18.3	ns	−0.18	−0.01	0.03	−0.12
	rs1327175	C<G	6.3	ns	−0.01	−0.21	0.16	−0.24
	rs2767565	A<G	18.7	ns	0.02	−0.00	−0.06	−0.06
	rs716461	A<G	28.3	ns	0.01	−0.05	0.10	0.08
RGS2	rs3767488	G<A	27.0	ns	0.02	0.03	–	–
	rs2746071	G<A	28.6	ns	0.03	−0.01	–	–
RGS4	rs10917670	A<G	43.0	ns	−0.10	−0.08	–	–
	rs951436	G<T	48.9	ns	0.09	0.12	–	–
	rs951439	A<G	42.8	ns	−0.10	−0.09	–	–
	rs2661319	A<G	49.6	ns	0.00	−0.15	–	–
RGS16	rs569790	A<G	36.1	0.0062	0.05	0.03	–	–
	rs610367	C<T	39.3	ns	0.08	−0.11	–	–

ns: Non-significant.

Multiplex genotyping assays were designed using the Sequenom MassARRAY Assay Design software Version 3.1 and performed with MassARRAY iPLEX (Sequenom Inc., San Diego, CA). Amplification reactions were based on the manufacturer's instructions with a modified protocol (Macgregor *et al.* 2008). Genotypes were assigned by using Typer Analyzer Version 4.0 software (Sequenom, San Diego, CA). Disagreements or unclear positioned dots produced by Typer Analyzer 4.0 in addition to all wells that had 50% or more failed SNPs were excluded from the analysis.

Several genotyping error checks were performed. Using the family data, Mendelian errors were checked. After these corrections, missingness per SNP was calculated. It appeared that genotyping was successful in more than 98% of the subjects for all SNPs with the exception of TPH2 rs1352251 (2.5% missing), COMT rs4680 (6.4% missing) and HTR2A rs6311 (17.9% missing). For these three SNPs, we checked whether the genotypes differed in a subsample of subjects (*N* = 282) who were genotyped for the same SNPs in another lab. Because this was not the case, we decided to analyse these SNPs as well. Hardy–Weinberg Equilibrium (HWE) was tested in Haploview (Barrett *et al.* 2005; Wigginton *et al.* 2005) resulting in four SNPs with a *P*-value below 0.01 (Table 1). These four SNPs were still included in the analyses, so that consistencies in results, e.g. more SNPs in one gene showing a significant result, were not overlooked.

## Results

### Factor analysis in total sample of adults

Table 2 shows the mean age and A/D scores at the five time points. The lower mean ages at 1991 and 1995 can be explained by the absence of parents and siblings (usually older than the twins) in these samples and by the ascertainment of mainly adolescent twins in the beginning. After 1993, older twins were also ascertained.

**Table 2:** Adults: Mean (SD) age and anxious depression (A/D) scores at the five time points in the total sample and in the genotyped sample

	Total sample						Genotyped sample					
		
	Men			Women			Men			Women		
				
	N	Age	A/D	N	Age	A/D	N	Age	A/D	N	Age	A/D
1991	642	19.9 (1.2)	4.9 (4.4)	833	19.8 (1.2)	6.7 (5.3)	63	20.0 (1.4)	4.7 (4.9)	114	19.9 (1.3)	7.4 (6.0)
1995	993	21.5 (2.2)	4.0 (3.9)	1275	21.5 (2.2)	6.1 (5.2)	151	21.7 (2.5)	3.9 (3.9)	246	21.3 (2.4)	6.6 (5.7)
1997	1458	26.9 (9.2)	4.1 (4.1)	2187	27.8 (9.5)	6.7 (5.4)	298	31.1 (12.2)	4.2 (4.3)	519	31.4 (11.5)	6.6 (5.7)
2000	1795	29.6 (9.6)	4.8 (4.6)	3447	30.5 (9.9)	7.2 (5.4)	311	33.8 (11.9)	4.2 (4.5)	603	33.1 (10.8)	7.2 (5.5)
2002	2859	41.5 (14.3)	4.8 (4.7)	4838	38.7 (13.1)	7.6 (5.8)	425	43.2 (13.9)	4.8 (5.0)	751	40.1 (12.8)	7.5 (5.8)

In the total sample, 60% of the twins and 53% of the siblings completed the A/D scale more than once. Parents were only invited once to participate. The phenotypic correlations across the five time points for A/D varied between 0.45 and 0.66 for men and between 0.48 and 0.70 for women (Table 3). The use of a one-factor model was supported as correlations were rather stable across occasions, showing only a slight decrease over time.

**Table 3:** Adults: Correlations between anxious depression (A/D) scores at the five time points. Below diagonal: men, above diagonal: women

	A/D 1991	A/D 1993	A/D 1997	A/D 2000	A/D 2002
A/D 1991		0.60	0.54	0.49	0.48
A/D 1993	0.54		0.67	0.60	0.55
A/D 1997	0.47	0.61		0.69	0.60
A/D 2000	0.49	0.56	0.62		0.70
A/D 2002	0.45	0.52	0.62	0.66	

The A/D factor explained 43%, 55%, 62%, 65% and 62% of the total variance of the five subsequent measurements in men and 45%, 59%, 64%, 73% and 62% in women. Even more important, the correlations between the latent factors were 0.69 and 0.70 for MZM and MZF twins and 0.31, 0.30 and 0.29 for DZM, DZF and DOS twins. A rough estimate of the heritability of the A/D factor, based on these correlations (Posthuma *et al.* 2003) equals 70%, which is substantially higher than the heritability estimates of around 45% for the individual A/D measures (Boomsma *et al.* 2000; Middeldorp *et al.* 2006a). This supports earlier analyses showing that an individual's stable vulnerability for A/D is largely influenced by genetic factors.

### Factor analysis in total sample of children

As expected, means for A/D hardly differ between boys and girls at age 7–12, but adolescent girls score higher than adolescent boys (Table 4). A/D scores also increase with age. As in the adult sample, A/D data were obtained at more than one point in time for 60% of the twins. Correlations for maternal and paternal A/D ratings from age 7 to 12 vary between 0.36 and 0.61 and between 0.39 and 0.62 for boys and girls, respectively (Table 5). Correlations between paternal and maternal ratings are similar to correlations between measures of the same rater across time. Correlations are clearly lower with self-report A/D measured during adolescence.

**Table 4:** Children: Mean (SD) A/D scores at the different ages in the total and the genotyped sample

		Total sample				Genotyped sample			
			
		Boys		Girls		Boys		Girls	
					
		N	A/D	N	A/D	N	A/D	N	A/D
Age 7	Mother	8950	2.2 (2.8)	9158	2.4 (2.9)	479	2.1 (2.5)	509	2.6 (3.0)
	Father	6609	1.7 (2.3)	6697	1.7 (2.4)	414	1.7 (2.2)	446	2.0 (2.8)
Age 10	Mother	5846	2.6 (3.2)	6208	2.7 (3.4)	468	2.5 (3.0)	480	3.1 (3.9)
	Father	4234	1.9 (2.6)	4444	2.0 (2.7)	399	2.0 (2.4)	389	2.1 (2.9)
Age 12	Mother	3938	2.3 (3.2)	4147	2.4 (3.1)	438	2.1 (2.6)	471	2.8 (3.5)
	Father	2941	1.8 (2.7)	3121	1.8 (2.6)	367	1.7 (2.3)	396	1.9 (3.0)
Age 14–18	Self	2319	3.1 (3.3)	2916	4.9 (4.5)	364	3.0 (3.3)	454	4.8 (4.3)

**Table 5:** Children: Correlations between the maternal (M), paternal (F) and self-report (S) anxious depression (A/D) scores across time. Below diagonal: men, above diagonal: women

		Age 7		Age 10		Age 12		Age 14–18
					
		M	F	M	F	M	F	S
Age 7	M		0.57	0.56	0.44	0.50	0.39	0.19
	F	0.59		0.39	0.53	0.37	0.48	0.17
Age 10	M	0.56	0.42		0.58	0.62	0.46	0.14
	F	0.44	0.53	0.60		0.45	0.56	0.23
Age 12	M	0.49	0.36	0.64	0.49		0.61	0.29
	F	0.42	0.49	0.51	0.60	0.61		0.25
Age 14–18	S	0.15	0.17	0.20	0.19	0.21	0.20	

In boys, the A/D factor explained 51%, 61% and 55% of the variance in maternal ratings, 46%, 56% and 53% in paternal ratings and 6% of the self-report. In girls, the A/D factor explained 52%, 57% and 55% of the variance in maternal ratings, 46%, 51%, 50% in maternal ratings and 9% of the self-report. More importantly, the MZM and MZF correlations for the latent factor were 0.77 and 0.74, respectively, and the DZM, DZF and DOS correlations were 0.40, 0.43 and 0.48, respectively. This yields a rough estimate of the heritability of the A/D factor of around 60%, which is similar to the heritability estimate at age 7, but substantially higher than the heritability estimates for the other ages (∼40%) (Boomsma *et al.* 2005).

### Association analyses

In adults, A/D means and standard deviations in the genotyped sample were comparable to those in the total sample of twins, sibling and parents, indicating that the genotyped sample is representative (Table 2). In the genotyped sample, 89% of the twins and 75% of the siblings completed more than one A/D survey.

For the power analyses in Mx, only the number of unique genotypes was considered. The total genotyped male and female sample contained 80% power to detect an additive genetic effect explaining at least 2.3% and 1.4%, respectively, of the variance with an *α* of 0.01. To compare, the same samples contained 80% power to detect an additive genetic effect explaining at least 3.1% and 1.9% in a *univariate* model. The subsample used for the analyses of the RGS SNPs contained 80% power to explain 3.6% and 2.5% of the variance. This would have been 5.0% and 3.4% of the variance in a *univariate* model.

The results of the association analyses are shown in Table 1. The lowest *p*-value was found for rs6265 in men *p* = 0.005). This is the val66met polymorphism in BDNF. In the male sample, the met allele was associated with lower A/D. Only one additional SNP, rs676643 in HTR1D, reached a *p*-value below 0.01 in men. No significant results were obtained in the female sample.

For children, A/D means and standard deviations in the genotyped sample were comparable to those in the total sample of twins, indicating that the genotyped sample is representative (Table 4). Longitudinal A/D data were available for more than one time point for 94% of the genotyped children.

Power analyses, based on the number of unique genotypes, showed that the power was 80% to find an effect explaining 3.1% of the variance in boys and girls with an alpha of 0.01. The same samples would have contained 80% power to explain 3.9% of the variance in a *univariate* model. However, in neither boys nor girls was a significant association with any of the SNPs and A/D observed.

## Discussion

This study investigated the influence of variation in genes involved in the serotonergic circuit or neurogenesis on anxiety and depression. Data were collected at four and five points across time in children and adults, respectively. The effect of the SNPs was modeled on a latent factor expressing the stability across A/D measures. On the basis of the twin correlations, heritability of the A/D factor appeared to be around 70% in the adults and 60% in the children, which is generally higher than the heritability based on the separate measures as the factor reflects an individual's stable vulnerability for A/D. Compared with most other candidate gene association studies, the present statistical power was adequate. Power analyses showed that the effect that could be detected ranged from 1.4% to 3.6% of the variance, which is considerably higher than for univariate models tested in samples of equal size.

Still, the findings do not support an influence of any of the SNPs on A/D. There were no significant effects in the children. In men, rs6265, the val66met polymorphism in BDNF, showed a significant effect with the met allele decreasing A/D scores. The direction of the effect was opposite to the male specific effect found in a meta-analysis investigating the association between the val66met polymorphism and major depression (Verhagen *et al.* 2010). It was in agreement with a meta-analysis of anxiety disorders and anxiety-related traits in which the met allele was related to lower neuroticism scores which did not test sex-specific effects (Frustaci *et al.* 2008). In this study, the (non-significant) effect in women was opposite to the effect in men. Overall, considering the discrepancies in results of the various studies, including ours, on the val66met polymorphism and anxiety and depression, there seems to be little evidence for a genuine association.

For the HTR1D SNP rs676643 an association with A/D was also shown (*p* value < 0.01) in the male sample. Given the number of tests combined with the lack of consistency in results (no effect in women nor in children), this result is probably as a result of chance.

The absence of an association between A/D and variation in serotonergic genes and BDNF is in agreement with genome-wide association (GWA) analyses of major depression whose results did not suggest an association with previously investigated genes (Muglia *et al.* 2008; Sullivan *et al.* 2009). In one of these studies, one SNP in BDNF and one SNP in TPH2 reached *p*-values, which were not corrected for multiple testing, of 0.05 and 0.03, respectively, in the single SNP analysis, but none of the genes showed significant gene-wide association (Muglia *et al.* 2008). Even a study that looked in more detail into the results of the second GWA study and applied a less stringent correction for multiple testing given the *a priori* higher chance to detect an effect, did not yield a significant effect for the genes investigated in this study (Bosker *et al.*, in press). Meta-analyses of the effect on major depression of the same SNPs in BDNF, COMT, HTR1A and HTR2A as in this study did not find an effect either (Levinson 2005; Lopez-Leon *et al.* 2008). Our result for COMT is not in agreement with the sex-specific effect suggested by Domschke *et al.* (2007), but further studies investigating this female-specific association have also shown inconsistent results (Hettema *et al.* 2008; Wray *et al.* 2008). No meta-analyses have been performed for TPH2, PlexinA2 and RGS. A review of the results for TPH2 concluded that results have been inconclusive so far (Zhang *et al.* 2004). The lack of association found in both children and adults suggests that TPH2 does not play a role in A/D either. There are several reasons for the discrepancies in results of the three studies investigating RGS2 and PlexinA2, such as differences in phenotypes (Leygraf *et al.* 2006; Wray *et al.* 2007), or differences in markers genotyped (Smoller *et al.* 2008). However, the positive results being caused by chance cannot be ruled out, despite the high quality of the studies.

Despite the lack of a positive result in our study, we emphasize that this design is valuable in future genome-wide association studies, especially the studies on developmental psychopathology. It is well known that the first GWA studies of psychiatric phenotypes were underpowered (Craddock *et al.* 2008). As most of the phenotypes have been measured repeatedly in the samples that will supply data for GWA studies of developmental phenotypes (e.g. Generation R, Avon Longitudinal Study of Parents and Children (ALSPAC) and the Young Netherlands Twin Register (Y-NTR) (Bartels *et al.* 2007;Golding *et al.* 2001; Jaddoe *et al.* 2008)) the combination of a longitudinal design and pooling the data in meta-analyses seems the most fruitful approach to increase the power.

Our study has a number of limitations. The YASR is not a frequently used measure of vulnerability for anxiety or depressive disorders in adults. However, earlier analyses in the same sample have shown that the association between Diagnostic and Statistical Manual of Mental Disorders (DSM-IV) diagnoses of depressive and anxiety disorders and the YASR scores is as strong as the association with neuroticism (Wilde 1970), a widely used indicator of an individual's vulnerability for anxiety and depression (Middeldorp *et al.* 2006b). Moreover, the YASR and the CBCL are part of the ASEBA, which makes the A/D scales of both questionnaires highly comparable and therefore very suitable given the purpose of this study to investigate whether the same SNPs influence A/D in children and adults.

The analyses were not corrected for population stratification. Population stratification can lead to false-positive as well as false-negative results if the subpopulations in the population differ in allele frequencies at a locus ánd in the trait means (Posthuma *et al.* 2004). Family data are suitable to test for population stratification by disentangling the SNP effect in a between-family and a within-family effect (Fulker *et al.* 1999; Medland & Neale 2010). The within-family effect is not confounded by population stratification as members from the same family arise from the same population. No significant difference between the between- and within-family effects indicates the absence of population stratification for that particular SNP. We have performed these analyses in the child and adult samples. Sex differences were not included in the model as the primary analyses did not suggest any sex-specific genetic effects. For none of the SNPs was the within-family effect significantly different from the between-family effect (no *p*-values below 0.01). Thus, it is highly unlikely that population stratification has led to any false-negative results.

Gene–environment interaction, haplotypes and recessive or dominant effects were not analysed in order to restrict the number of tests. A simulation study showed that it is statistically unlikely to find an interaction effect with an environmental risk factor with a relatively high exposure rate, such as life events, if a main effect of the genetic variant is not found (Munafo *et al.* 2009). As several studies have indicated a recessive effect of the met allele of the rs6265 BDNF SNP (Chen *et al.* 2006; Montag *et al.* 2009; Verhagen *et al.* 2010), the association analyses were repeated testing the effect of the met–met genotype versus the effect of the met–val and val–val genotypes. No significant results were found in the adult and children's male and female samples.

This study did not cover all genes involved in the serotonergic system or in neurogenesis, but was restricted to genetic variants that seemed most promising at that time. The recent literature does not indicate that other genetic variants in these pathways are more likely to show an effect (Lopez-Leon *et al.* 2008). One exception is the serotonin transporter gene length polymorphism (5-HTTLPR). However, we previously showed no effect of 5-HTTLPR on neuroticism, anxiety, depression and major depressive disorder in a large, partly overlapping, adult sample (Middeldorp *et al.* 2007).

To conclude, this longitudinal study in children and adults found no association of SNPs in the serotonergic system or in core regulators of neurogenesis with A/D. This is in line with previous candidate gene studies as well as the more recent GWA studies. Currently, convincing evidence for a role for genetic variation in these pathways in the development of anxiety and depression in children or adults is still missing.

## Supporting Information

**Figure S1:** LD plots of PLXNA2, BDNF and TPH2 indicating *D*' between the SNPs.

As a service to our authors and readers, this journal provides supporting information supplied by the authors. Such materials are peer-reviewed and may be re-organized for online delivery, but are not copy-edited or typeset. Technical support issues arising from supporting information (other than missing files) should be addressed to the authors.
