# Supplementary material for: Anxiety and depression in children and adults: influence of serotonergic and neurotrophic genes?
Source: Genes Brain Behav. 2010 Oct;9(7):808–16. doi: 10.1111/j.1601-183X.2010.00619.x (PMC3151552; doi:10.1111/j.1601-183X.2010.00619.x)
Supplement: Supplementary file 1 [file gbb0009-0808-SD1.doc]

**Supplementary figure 1: LD plots of PLXNA2, BDNF and TPH2**

PLXNA2 BDNF

TPH2

**Legends**

Supplementary Figure 1: LD plots of PLXNA2, BDNF and TPH2 indicating D’ between the SNPs.
